# Reducing neonatal mortality associated with preterm birth: gaps in knowledge of the impact of antenatal corticosteroids on preterm birth outcomes in low-middle income countries

**DOI:** 10.1186/s12978-016-0180-6

**Published:** 2016-05-24

**Authors:** Elizabeth M. McClure, Robert L. Goldenberg, Alan H. Jobe, Menachem Miodovnik, Marion Koso-Thomas, Pierre Buekens, Jose Belizan, Fernando Althabe

**Affiliations:** Social, Statistical and Environmental Health Sciences, RTI International, 3040 Cornwallis Road, Durham, NC USA; Department of Obstetrics and Gynecology, Columbia University, New York, NY USA; Cincinnati Children’s Hospital, Cincinnati, OH USA; Eunice Kennedy Shriver National Institute of Child and Human Development, Bethesda, MD USA; Tulane University School of Tropical Medicine and Hygiene, New Orleans, LA USA; Institute for Clinical Effectiveness and Health Policy, Buenos Aires, Argentina

## Abstract

The Global Network’s Antenatal Corticosteroids Trial (ACT), was a multi-country, cluster-randomized trial to improve appropriate use of antenatal corticosteroids (ACS) in low-resource settings in low-middle income countries (LMIC). ACT substantially increased ACS use in the intervention clusters, but the intervention failed to show benefit in the targeted < 5th percentile birth weight infants and was associated with increased neonatal mortality and stillbirth in the overall population. In this issue are six papers which are secondary analyses related to ACT that explore potential reasons for the increase in adverse outcomes overall, as well as site differences in outcomes. The African sites appeared to have increased neonatal mortality in the intervention clusters while the Guatemalan site had a significant reduction in neonatal mortality, perhaps related to a combination of ACS and improving obstetric care in the intervention clusters. Maternal and neonatal infections were increased in the intervention clusters across all sites and increased infections are a possible partial explanation for the increase in neonatal mortality and stillbirth in the intervention clusters, especially in the African sites. The analyses presented here provide guidance for future ACS trials in LMIC. These include having accurate gestational age dating of study subjects and having care givers who can diagnose conditions leading to preterm birth and predict which women likely will deliver in the next 7 days. All study subjects should be followed through delivery and the neonatal period, regardless of when they deliver. Clearly defined measures of maternal and neonatal infection should be utilized. Trials in low income country facilities including clinics and those without newborn intensive care seem to be of the highest priority.

## Background

Preterm birth is now considered the most common cause of neonatal mortality worldwide [1]. To reduce neonatal mortality and morbidity associated with preterm birth, antenatal corticosteroids (ACS) are commonly used in women at risk to deliver preterm in both high-income countries (HIC) and middle-income countries (MIC) [2, 3]. To date, the efficacy of ACS has been studied in HIC and some MIC settings in 26 randomized trials and summarized in numerous meta-analyses [4–9]. Overall, when ACS are given to the mother in those settings between 24 and 34 weeks gestation, at 12–24 hours prior to delivery and the delivery occurs within 7 days, there is a 31 % reduction in the neonatal mortality rate (NMR) as a result of reduced risks of respiratory distress syndrome, intraventricular hemorrhage, necrotizing enterocolitis, and other pleotropic effects that improve infant outcomes [4, 6, 7]. Although the ACS are generally considered safe for the mother and newborn, a slight increase risk in neonatal mortality among women who receive ACS and delivered at term was reported in two trials [6]. Moreover, one meta-analysis of randomized trials of infants whose mothers received ACS but delivered more than one week after ACS administration showed higher perinatal mortality rates and more perinatal infections than the controls [9].

To address the lack of information on the impact of ACS use in low and middle income countries (LMIC), the NICHD Global Network designed a cluster randomized trial, the Antenatal Corticosteroids Trial (ACT), in Zambia, Kenya, Pakistan, India, Argentina and Guatemala to assess the effects of an intervention to increase use of ACS through training birth attendants to identify pregnant women at high risk of preterm birth and providing ACS kits [10]. Even though ACS use increased from about 10 % in the control clusters to 46 % in the intervention clusters, not only did NMR *not* decrease among low birth weight (<5th percentile) infants as hypothesized, but the NMR and stillbirth rates also increased overall, an unanticipated finding [11].

We performed a series of secondary analyses in order to better understand the failure of the ACT intervention to reduce neonatal mortality in the target group and the increased overall neonatal mortality and stillbirth rates. We also reviewed prior research on ACS, and held a meeting with experts from the National Institutes of Health, World Health Organization, Bill and Melinda Gates Foundation, USAID and other investigators studying corticosteroids to present the secondary ACT findings. Here we summarize the secondary analyses.

### Secondary analyses

A series of secondary analyses further explored the reasons for the unexpected overall increases in neonatal mortality and stillbirth, the possible impact of maternal and newborn infection on these outcomes, and the site differences [12–16]. We include a case study from Guatemala, where a significant improvement in neonatal mortality was observed in the target group. Furthermore, we examined baseline rates of neonatal infection across Global Network sites [17]. The analyses suggest that a potential explanation for the increase in both neonatal mortality and stillbirth in the intervention clusters was an increase in neonatal and maternal infections. There were also worse outcomes associated with the intervention in the African sites but an apparent reduction in neonatal mortality among the target < 5^th^ percentile birth weight infants in the Guatemala site. These results presented in this journal suggest that the decrease in neonatal mortality in Guatemala may have been related to the combination of improved medical care plus ACS use. Across all sites, the increase in neonatal mortality and stillbirth in the treatment clusters overall may have been related to increased maternal and neonatal infection associated with the intervention. However, no definitive reasons for the increases in either neonatal mortality or stillbirth have been identified. Equally as important, we emphasize that there was no decrease in neonatal mortality in the targeted <5^th^ percentile infants. These hypothesis-generating secondary analyses suggest that future research should focus on neonatal and maternal infectious outcomes as well as the importance of collecting trial outcomes through delivery and the neonatal period among all women and their fetuses and neonates who receive ACS and their controls.

### ACT in the context of other ACS research

A cautionary observation about ACS research is that most of the trials to establish efficacy were conducted between the 1970’s and the early 1990’s, more than 25 years ago. As time elapses from original research and practice, there is increased likelihood for the results to have changed [18, 19]. Thus, careful assessment of older studies in the context of new research is warranted.

The ACT study had several characteristics which distinguished it from the individually randomized trials performed in HICs and MICs from 1972 through the 2000’s. First, the initial trials were conducted in hospitals with good obstetric and neonatal care, while ACT included deliveries within defined geographic areas among an entire population, whether deliveries were in the homes, clinics or lower level hospitals. Few, if any, of the hospital births in ACT were in facilities that approached the level of care available for the previous trials. Second, because ultrasound was rarely available and the gestational age dating unreliable, the ACT study used a surrogate measure for preterm delivery, births at <5^th^ percentile of birth weight adjusted for the population at each participating site. Third, even though the ACT supervisors trained health workers on the conditions associated with preterm birth including preterm labor, membrane rupture, significant bleeding and preeclampsia that prompted ACS administration, these providers – often community health workers or traditional birth attendants – generally had little experience in diagnosing those conditions. Fourth, at some of the ACT sites, the women were more likely than women in the prior trials to carry various infectious organisms and have poor nutritional status. Women and newborns in the entire ACT population for both arms of the trial were followed to 42 days after birth and thus neonatal outcomes were available for the entire population of deliveries at each site. Follow-up to this extent was rarely performed in prior trials. Finally, the ACT study included nearly 100,000 deliveries in the clusters of which more than 6,000 mothers in the intervention clusters received ACS, while the sum of those enrolled and randomized in the initial trials of a single course of ACS was approximately 4,000. The small but significant increase in NMR observed in the ACT results would not have been detected in the smaller previous studies with lower baseline risks.

Another important strength of ACT was the attempt to collect outcome data on everyone in the trial whether they were delivered in a hospital or not through 42 days post-delivery. In reviewing previous research of ACS, the follow-up of those women and newborns who delivered outside the expected window of ACS benefit or outside the preterm period was often inadequate. In most cases, data were available only to hospital discharge; outcomes among infants in the studies after discharge for most ACS research were usually not available. A subgroup analysis of the 2006 Cochrane review of 18 studies evaluating neonatal mortality, found that that only three of the studies included results at gestational age >36 weeks [6]. Thus of the 18 trials including near 4,000 women, we do not know the proportion of women overall who eventually delivered at term. Interestingly, of the 613 newborns for which this type of data were available, the neonatal mortality rate was increased 2 to 3 fold. Another review asked the question about outcomes in women who delivered more than seven days after receiving ACS [9]. In this meta-analysis, only seven studies had data to answer this question. In the analysis limited to those studies, both perinatal mortality and chorioamnionitis were increased among maternal/infant dyads who received ACS. The ACT results, which showed a small increase in neonatal mortality and stillbirths concentrated in the heavier and more mature infants and fetuses and a possible increase in maternal and neonatal infections, are consistent with the available results from individually randomized trials in HIC.

Another difference among trials is the choice of corticosteroids - betamethasone or dexamethasone. Several trials comparing these corticosteroids have shown that betamethasone may be more efficacious than dexamethasone, although in a meta-analyses, differences were not statistically different [20, 21]. The formulation of betamethasone phosphate and betamethasone acetate initially used by Liggins and Howie has been more frequently been used in HIC research [22]. Dexamethasone, which is the WHO recommended drug and is more widely available in LMIC likely due to its stability and lower cost compared to betamethasone, was used for ACT [23, 24]. In the meta-analyses, dexamethasone was associated with maternal infection (although non-significant) and there may be subtle but important differences in the effect of these different drugs. The optimal dose for dexamethasone is unknown [6, 21].

### Gaps in research

Given the results from ACT and other trials, it is clear that a number of important research questions remain regarding ACS research, especially in low-resource settings. First, since it is likely that only pregnancies at risk of preterm delivery are likely to benefit from ACS, it is crucial to have accurate gestational age dating in order to enroll subjects within the desired treatment window. Second, it is also essential to have qualified obstetric providers make the judgment about which patients are likely to deliver within the one week period of maximal benefit. Third, it is necessary to follow each enrolled patient and her newborn to delivery and for at least 28 days after birth. Following only those who deliver within the preterm period or to hospital discharge will not provide a full understanding of the benefits and risks of ACS. Finally, one must determine the appropriate level of care where research is warranted. A trial conducted in a LMIC probably should not randomize women delivering in facilities similar to those in which the high-income studies were undertaken, since there is little reason to believe that in those facilities, benefits would not occur. Also, given the difficulties the ACT teams encountered in getting ACS to the appropriate women who have their suspected preterm births at home as well as the necessary follow-up care, we do not believe that another ACS trial involving home births is appropriate at this time.

There is a large movement throughout LMIC to shift births into clinics and lower level or district hospitals, generally without newborn special care [25]. Therefore, we believe the most important question to answer right now is whether ACS can be given to the appropriate women and whether ACS reduce neonatal mortality in those settings [26]. Any study done on ACS in low-income settings must evaluate the mothers, fetuses and newborns for infection to evaluate the safety of the intervention. In summary, research questions that may inform future programs to roll-out ACS in these settings include the following:

1. Gestational age assessment: What is the best way to determine gestational age and which outcomes associated with ACS use are impacted in different gestational age windows?
2. Risk population: Which factors predict preterm delivery in LMIC and what is the best way to ensure that providers can identify signs of risk?
3. Obstetric and neonatal care: What is the impact of ACS when administered to women delivering at different levels of obstetric and neonatal care?
4. Infectious outcomes: What maternal and newborn infectious outcomes are associated with ACS?

## Conclusions

The ACT findings have important programmatic implications for many international global health agencies, foundations and individual countries planning on scaling up the use of ACS. The lack of benefit in the targeted neonates and the increased stillbirths and neonatal deaths overall were unexpected. Our attempts to understand these outcomes in secondary analyses of the ACT data provided clues but no clear answers. These analyses do however provide guidance for future ACS trials in LMIC. These include having gestational age dating of study subjects and having care givers who can diagnose conditions leading to preterm birth and predict with some degree of accuracy which women are likely to deliver within the next seven days. Finally, all subjects entered into the trial should be followed through delivery and the neonatal period, regardless of whether they deliver preterm or at term. Clearly defined measures of maternal and neonatal infection should be determined prior to any study and be included as an outcome. Trials in low income country facilities including clinics and those without newborn intensive care seem to be of the highest priority.

## References

[1] Lawn JE, Kinney M (2014). Preterm birth: now the leading cause of child death worldwide. Sci Transl Med.

[2] Darmstadt G, Bhutta Z, Cousens S, Adam T, Walker N, de Bernis L (2005). Evidence-based, cost-effective interventions: how many newborn babies can we save?. Lancet.

[3] Vogel JP, Souza JP, Gülmezoglu AM, Mori R, Lumbiganon P, Qureshi Z (2014). WHO multi-country survey on maternal and newborn health research network. Use of antenatal corticosteroids and tocolytic drugs in preterm births in 29 countries: an analysis of the WHO multicountry survey on maternal and newborn health. Lancet.

[4] Liggins Institute. Antenatal corticosteroids given to women prior to birth to improve fetal, infant, child and maternal health. Available at http://www.ligginstrials.org/ANC_CPG/downloads/Antenatal_Corticosteroid_Clinical_Practice_Guidelines.pdf (Accessed 8 Mar 2016)

[5] National Institutes of Health. The effect of corticosteroids for fetal maturation on perinatal outcomes. Consensus Development Conference Statement, Feb 28–March 2, 1994. http://consensus.nih.gov/1994/1994AntenatalSteroidPerinatal095html.htm (Accessed 1 June 2014).

[6] Roberts D, Dalziel S. Antenatal corticosteroids for accelerating fetal lung maturation for women at risk of preterm birth. Cochrane Database Syst Rev. 2006;3:CD004454. 10.1002/14651858.CD004454.pub2 16856047

[7] World Health Organization. WHO recommendations on interventions to improve preterm birth outcomes. http://apps.who.int/rhl/guidelines/preterm-birth-guideline/en/. (Accessed 8 Mar 2016) 26447264

[8] Mwansa-Kambafwile J, Cousens S, Hansen T, Lawn J (2010). Antenatal steroids in preterm labour for the prevention of neonatal deaths due to complications of preterm birth. Int J Epidemiol.

[9] McLaughlin KJ, Crowther CA, Walker N, Harding JE (2003). Effects of a single course of corticosteroids given more than 7 days before birth: a systematic review. Aust N Z J Obstet Gynaecol.

[10] Althabe F, Belizán JM, Mazzoni A (2012). Antenatal corticosteroids trial in preterm births to increase neonatal survival in developing countries: study protocol. Reprod Health.

[11] Althabe F, Belizán JM, McClure EM, Hemingway-Foday J, Berrueta M, Mazzoni A (2015). A population-based, multifaceted strategy to implement antenatal corticosteroid treatment versus standard care for the reduction of neonatal mortality due to preterm birth in low-income and middle-income countries: the ACT cluster-randomised trial. Lancet.

[12] Althabe F, Thorsten VR, Klein K, et al. The Antenatal Corticosteroids Trial (ACT)’s explanations for neonatal mortality - a secondary analysis. Reprod Health. (in press) 10.1186/s12978-016-0175-3 PMC4878056 27220987

[13] Althabe F, Thorsten VR, Klein K, et al. The Antenatal Corticosteroids Trial (ACT): A secondary analysis to explore site differences in a multi-country trial. Reprod Health. (in press) 10.1186/s12978-016-0179-z PMC4878061 27221319

[14] Garces A, McClure EM, Figueroa L, et al. A multi-faceted intervention including antenatal corticosteroids to reduce neonatal mortality associated with preterm birth: A case study from the Guatemalan Western Highlands. Reprod Health. (in press) 10.1186/s12978-016-0178-0 PMC4877983 27221237

[15] Goldenberg RL, Thorsten VR, Althabe F, et al. The Global Network Antenatal Corticosteroids Trial: Impact on Stillbirth. Reprod Health. (in press) 10.1186/s12978-016-0174-4 PMC4891888 27255082

[16] Berrueta M, Hemingway-Foday, J, et al. Antenatal Corticosteroids: Barriers and facilitators to use among 7 low-middle income countries. Reprod Health. (in press) 10.1186/s12978-016-0176-2 PMC4882797 27228986

[17] Hibberd PL, Hansen NI, Wang M, et al. Trends in the Incidence of Possible Severe Bacterial Infection and Case Fatality Rates in Rural communities in Sub-Saharan Africa, South Asia and Latin America, 2010–2013: A multicenter prospective cohort study. Reprod Health. (in press) 10.1186/s12978-016-0177-1 PMC4877736 27221099

[18] Anyaegbunam WI, Adetona AB (1997). Use of antenatal corticosteroids for fetal maturation in preterm infants. Am Fam Physician.

[19] Spinillo A, Capuzzo E, Ometto A, Stronati M, Baltaro F, Iasci A (1995). Value of antenatal corticosteroid therapy in preterm birth. Early Hum Dev.

[20] Senat MV, Minoui S, Multon O, Fernandez H, Frydman R, Ville Y (1998). Effect of dexamethasone and betamethasone on fetal heart rate variability in preterm labour: a randomised study. Br J Obstet Gynaecol.

[21] Brownfoot FC, Gagliardi DI, Bain E, Middleton P, Crowther CA (2013). Different corticosteroids and regimens for accelerating fetal lung maturation for women at risk of preterm birth. Cochrane Database Syst Rev.

[22] Liggins GC, Howie RN (1972). A controlled trial of antepartum glucocorticoid treatment for prevention of the respiratory distress syndrome in premature infants. Pediatrics.

[23] The selection and use of essential medicines: report of the WHO Expert Committee, 2013 (including the 18th WHO Model List of Essential Medicines and the 4th WHO Model List of Essentials Medicines for Children). Geneva: World Health Organization; 2013.

[24] Hofmeyr GJ. Antenatal corticosteroids for women at risk of preterm birth: RHL commentary (last revised: 2 February 2009). The WHO Reproductive Health Library. Geneva: World Health Organization.

[25] Moyer CA, Mustafa A (2013). Drivers and deterrents of facility delivery in sub-Saharan Africa: a systematic review. Reprod Health.

[26] Hodgins S (2014). Caution on corticosteroids for preterm delivery: learning from missteps. Glob Health Sci Pract.

